# The establishment of the Chiropractic & Osteopathic College of Australasia in Queensland (1996–2002)

**DOI:** 10.1186/1746-1340-13-3

**Published:** 2005-04-11

**Authors:** Bruce F Walker

**Affiliations:** 1 School of Medicine, James Cook University, Townsville, Queensland, Australia

**Keywords:** Chiropractic, osteopathy, continuing education, vocational education, evidence-based practice, Queensland

## Abstract

**Introduction:**

For chiropractors and osteopaths after graduation, the learning process continues by way of experience and continuing education (CE). The provision of CE and other vocational services in Queensland between 1996 and 2002 is the subject of this paper.

**Methods:**

The Chiropractic & Osteopathic College of Australasia (COCA) implemented a plan, which involved continuing education, with speakers from a broad variety of health provider areas; and the introduction of the concepts of evidence-based practice. The plan also involved building membership.

**Results:**

Membership of COCA in Queensland grew from 3 in June 1996 to 167 in 2002. There were a total of 25 COCA symposia in the same period. Evidence-based health care was introduced and attendees were generally satisfied with the conferences.

**Discussion:**

The development of a vocational body (COCA) for chiropractors and osteopaths in Queensland was achieved. Registrants in the field have supported an organisation that concentrates on the vocational aspects of their practice.

## Introduction

Chiropractic and Osteopathy are complementary health professions that enjoy Government imprimatur to the extent that they have Registration Boards in every jurisdiction and a National Uniform Code of Conduct [1]. Also, third party payers such as private health funds, workers compensation authorities and the Department of Veterans Affairs recognise both professions and fund treatment provided by approved chiropractors or osteopaths [2,3].

The training of chiropractors is by way of degree courses at Macquarie University (New South Wales [NSW]), Royal Melbourne Institute of Technology-RMIT University (Victoria) and a new program at Murdoch University in Perth (Western Australia). For osteopaths there are undergraduate courses at Victoria University of Technology, RMIT University (Victoria) and the University of Western Sydney (NSW) [4]. Importantly, there is no undergraduate program for these professions in Queensland.

After graduation the learning process usually continues by way of an experiential process and also (but not always) from continuing education. The provision of continuing education and other vocational services to chiropractors and osteopaths in Queensland between 1996 and 2002 is the subject of this paper.

To maintain Registration, continuing education for chiropractors and osteopaths is not compulsory in Australia as it is the USA. However, it is compulsory to maintain provider status with the Department of Veterans Affairs (DVA) [2]. As DVA income represents only a relatively small percentage of revenue for chiropractors and osteopaths there is no real financial or statutory imperative to participate in continuing education.

Before arguing for continuing education one must ask a preliminary question regarding its validity. To do this a MEDLINE literature search was conducted from 1980 until 2002, using the key indexing words of "continuing medical education", "chiropractic", "osteopathy", "educational intervention", "clinical audits", "performance indicator", "competency", "patient outcome" and "validity".

A search was also conducted of the literature indexing system for chiropractors and osteopaths known as MANTIS [5]. From this search a key paper by Werth [6] identified a paucity of information on the subject of continuing education concerning chiropractors and osteopaths; this paper reviews and relies on the medical and para-medical literature to review continuing education from a number of different perspectives including continuing education definition, needs assessment, evaluation and compulsory participation. Two significant issues arising from this paper are practitioner performance and health care outcomes after the administration of continuing education.

Davis et al assessed these issues in a review of 50 randomised controlled trials [7]. The conclusion reached by the authors was there is evidence for changes in practitioner performance from continuing education but very little for improved patient outcome. A later paper by the same authors further reviewed studies that met the following criteria: randomized controlled trials of education strategies or interventions that objectively assessed physician performance and/or health care outcomes [8]. These intervention strategies included (alone and in combination) educational materials, formal continuing medical education (CME) activities, outreach visits such as academic detailing, opinion leaders, patient-mediated strategies, audit with feedback, and reminders.

They found 99 trials, containing 160 interventions. Almost two thirds of the interventions (101 of 160) displayed an improvement in at least one major outcome measure: 70% demonstrated a change in physician performance, and 48% of interventions aimed at health care outcomes produced a positive change. Effective change strategies included reminders, patient-mediated interventions, outreach visits, opinion leaders, and multifaceted activities. Audit with feedback and educational materials were less effective, and formal CME conferences or activities, without enabling or practice-reinforcing strategies, had relatively little impact.

Langworthy, in the only published study of clinical audit in chiropractic, concluding that a voluntary national audit scheme succeeded in raising awareness and standards of clinical practice [9]. Mugford *et al.*'s review of 36 studies of the use of statistical information from audit or practice reviews suggest that it is most likely to affect practice when the recipients have already agreed to review that practice [10]. Cantillon and Jones's review of CME in general practice found 18 evaluations of audits with educational interventions, of which 17 showed a positive influence on doctor behaviour [11]. A Cochrane review has concluded that audit and feedback may be effective in improving the practice of healthcare professionals, especially prescribing [12].

Therefore, it appears that evidence for continuing education in achieving a positive change in practitioner performance and health care outcomes is mixed with some evidence for specific styles of continuing education. On balance, it is plausible to argue that quality continuing education has a general beneficial effect.

The advent of evidence-based health care has increased the demand on health care providers of all persuasions to base their decisions and actions on the best possible evidence. The ability to receive or track down, critically appraise (for its validity and utility), and incorporate a rapidly growing body of evidence into one's clinical practice has been the "mantra" of the past decade [13].

Within the State of Queensland prior to 1996 practising chiropractors and osteopaths had some opportunities to participate in scientific forums specifically designed to improve their information gathering, clinical, scientific appraisal and bio-ethical skills. Dr. Keith Charlton principally developed this with the advent of the Brisbane Spinal Studies Group. However, when Dr. Charlton left for the United Kingdom there was an apparent lull.

Professional associations and their subsidiaries provided much of the continuing education in Queensland prior to 1996. The major associations then were the Chiropractors Association of Australia [14] (CAA) (and its antecedents) and the Australian Osteopathic Association [15] (AOA). It can be argued that these two National organisations are in many ways the equivalent of trade unions. They aim to represent the professions in every respect and to the best of their ability. In the three decades prior to 1996, important political issues such as Government Registration and third party payer acceptance privileges consumed much of these associations' time. Skilled chiropractors and osteopaths who gained political experience "on the job" have generally led both associations.

Apart from their political agenda both associations have historically provided annual and occasional conferences, educational seminars and also respective journals newsletters for their members. These have assisted knowledge advancement. Nevertheless, it was observed by the Chiropractic & Osteopathic College of Australasia (COCA) [16] Executive of the day that there was an educational and vocational hiatus for both professions. It was thought that this was because the associations were not providing enough "best practice" continuing education.

COCA determined that if chiropractors and osteopaths were to progress in the information and evidence-based age they would need access to high level material prepared and delivered by the best mentors available. In the mid 1990's it appeared that the associations (CAA and AOA) were strongly interested in the political agenda of the day and accordingly there appeared to be room for another group or professional body to provide these educational and other vocational services to chiropractors and osteopaths in Queensland.

The identification of this need led to the expansion of the COCA from a predominantly Victorian based organisation into the State of Queensland and later nationally. The objective of this expansion was to develop, provide and foster quality vocational and educational services for chiropractors and osteopaths in Queensland and other States, with the ultimate goal of improving the public health.

## Methods

The national Executive of COCA reviewed a draft plan to attain these objectives. This plan involved was formulated by the author and involved:

### 1. Commencing continuing education in Townsville (a regional city in North Queensland) and if successful expand to Brisbane the State's capital

This required some degree of "faith" on behalf of the COCA Executive, as the proposal was to run at a financial deficit (loss leader) for 2 to 3 years in both locations to encourage participation and COCA membership. It was a contention that once seminar attendances and membership had grown to a critical level, more realistic fees could be charged.

### 2. Organising conferences and offering the best available speakers from a broad variety of health provider areas

This decision was based on the notion that there was a wide-spread intellectual isolation of chiropractors and osteopaths in practice. In addition there were few opportunities for chiropractors and osteopaths to practice in multi-disciplinary environments. There was also a widespread feeling within the Executive of the day that University under-graduate education did not expose students to any training in hospitals where they would be likely to interact with a wide group of health professionals and also see very unwell patients. Accordingly, it was identified that speakers from other health fields would provide this initial interaction missing from the chiropractors and osteopaths professional training and life.

### 3. Introducing speakers who were chiropractors and osteopaths undertaking research at a high level and expose participants to this research

The modus operandi here was not to expose registrants to just research results but also the rigours of the methodology involved in such research. It was a goal that this exposure would give participants a better understanding of the research process.

### 4. Introducing the concepts of Evidence-based practice

Patient care is often outmoded because health providers of all persuasions lack awareness about important advances in their particular discipline and or general medical knowledge. One method of keeping abreast of current knowledge is reading journals. It is a popular method of staying informed, and is particularly suited to Queensland because of the vast size of the State and its decentralised structure.

However, it is one thing to read a journal article, it is another to critically review it. It was therefore considered important to continue the work of the Brisbane Spinal Studies Group by enhancing literature review skills for chiropractors and osteopaths in Queensland. Using this strategy COCA aimed at enhancing the efficiency and effectiveness of journal reading and provide sufficient skills to assess the relevance, validity, and clinical application of new knowledge.

### 5. Building COCA membership

All chiropractors and osteopaths in Queensland were offered membership of COCA. COCA reasoned that in order to build membership it was necessary to develop "ownership" in the concepts of a vocational College. The Executive took the view that it should model COCA along the lines of the Royal Australian College of General Practitioners (RACGP) [17]. A College of this nature has many objectives other than providing continuing education. It was therefore important for COCA to define a Mission Statement and Objectives over time. Many other benefits potentially accrue to members and the public if a College of this nature flourishes, particularly if such an organisation is built on a foundation of knowledge advancement, science, ethics, and the public health. Another benefit of building membership in Queensland was to recruit other practitioners to assist with COCA's activities.

### 6. Assessment of outcomes between 1996 and 2002

COCA assessed its continuing education in several ways. Initially, by surveying registrants after seminars and conferences, and then using this data and other pertinent educational material to develop guidelines for continuing education. As the organization expanded examining growth in COCA membership also became an important outcome measure. Where appropriate descriptive statistics of these measures were generated. Another outcome measure was whether COCA could establish itself as a stakeholder representing both professions on issues where there was a synergy with its stated objectives. This meant lobbying Government and other bodies about such issues.

## Results

### 1. Commencing in Townsville

A financial commitment was given by COCA in 1996 to commence operations in Queensland. These operations were based in Townsville and handled by Past President of COCA, Dr. Bruce Walker.

### 2,3. Conferences and speakers

The first conference was held in Townsville in June 1996. This conference set the scene for COCA in Townsville; it was multi-disciplinary with two medical specialists, one General Practitioner, 7 chiropractors and one chiropractor/histo-pathologist. Later that year another multi-disciplinary seminar was held, this one being held in conjunction with the James Cook University Chiropractic Research Fund. In 1997 the main conference was "An overview of orthopaedic surgery" delivered by Surgical Registrar and chiropractor Dr. David de la Harpe. Dr de la Harpe later specialised in Spine Surgery and is currently COCA Patron. A "meet and greet" for new members was held for new members in October 1997. On this informal occasion the guest speaker was Dr. R Jackson, from the Tennessee Chiropractic Licensing Board who spoke about Chiropractic in the USA and in particular on compulsory continuing education.

In 1998 buoyed by the success of the Townsville events, the first Brisbane Conference was approved by COCA and organised for March 1998. The seminar was strong on clinical science and in particular focussed on the reliability of clinical instruments of measurement. In the same month another Townsville seminar was held with a multi-disciplinary group of speakers. In July 1998 a follow up conference in Brisbane featuring Dr. de la Harpe was held and later that year in November another Brisbane conference was conducted. Thereafter there have been regular conferences and seminars in both Brisbane and Townsville. A one-off conference was also held on the Gold Coast.

In 2000 COCA gained the assistance of Drs. Ken Lorme (KL) and Jo-Anne Maire (JM). These two chiropractors (both with post-graduate degrees) assisted by taking over the conduct of COCA conferences in Brisbane (KL) and preparing a clinical audit program for members (JM). There was a total of 25 conferences or seminars organised by COCA between June 1996 and June 2002.

### 4. Evidence-based health care

At the initial COCA conference in Townsville participants were introduced to the concept of evidence-based health care. This was achieved by posing a clinical question "Does scoliosis predispose to back pain?" Then 3 key papers were presented and critically reviewed (by the author) demonstrating how to derive an answer to such a clinical question. A workshop conducted at the July 1998 Brisbane Conference presented similar work and using a checklist participants reviewed a chiropractic paper on infantile colic. The conclusion reached (after critical review by the group) was that the journal authors' conclusion was not supported by the study as published. It was for many their first systematic review of a journal article. COCA members are now regularly exposed to the concepts of evidence based health care.

### 5. Building COCA membership

The membership of COCA in Queensland grew from 3 in June 1996 to 167 in 2002. This growth must be seen as an endorsement by practitioners on the ground in Queensland as there were only about 400 registered chiropractors and osteopaths domiciled and working in the State at the time.

Queensland chiropractors and osteopaths often have geographical limitations placed on their continuing education ("The tyranny of distance"). As a consequence COCA felt it was important for these and other regional, rural and remote members to have access to both distance learning and a library. Distance learning is now an integral part of the benefits of COCA membership; further a special benefit for members is access to the RACGP library. Such access is crucial to Queensland members.

Other tangible benefits for members include regular multi-disciplinary continuing education in a variety of formats including distance learning at an affordable price, access to professional indemnity insurance, a journal (Australasian Chiropractic and Osteopathy), a regular newsletter (COCA News) and belonging to an organisation that has knowledge advancement, science, ethics and public health as its main objectives [16].

### 6. Other outcomes

The satisfaction of attendees at COCA conferences was measured using an optional "Exit questionnaire". In order to evaluate this variable a convenience sample of 181 was supplied by COCA secretariat. It appears from close review that some returns are missing. Also the response rate was not calculable because of a loss of COCA data at Secretariat level. Therefore the following results are only indicative for those returns processed and cannot be generalised to the entire population of attendees.

The first 2 questions of the survey ask "What were the best aspects of the seminar?" and "What were the worst aspects of the seminar?" A review of the answers to these questions shows that for question 1, there was a mean of 2.4 out of a possible 3 (sum total = 431) and for question 2, the mean was 0.71 out of 3 (sum total 127). These results show a substantially greater number of "best aspects" than "worst aspects".

The third question asks "Was the seminar value for money?", 175 (96.7%) answered "yes", 1 (0.6%) answered "no" and 5 were missing.

The fourth question asked respondents to rate the seminar on a Likert scale out of 7. With 1 being "very poor" and 7 being "very good". The mean rating for all surveys from all conferences was 6.1 (Range 3 to 7), missing 3.

## Discussion

The development of a vocational body (COCA) for chiropractors and osteopaths in Queensland is now a reality. Registrants in the field have supported ("with their feet") the notion of a body that delivers continuing education at an affordable cost and also an organisation that concentrates on the vocational aspects of their practice with a scientific and ethical focus. COCA's objectives are set out and are on the public record [16] and all applicants for membership sign their acceptance of the COCA Code of Ethics [16].

COCA has dedicated materials and resources used in its activities, to serve Queensland members and non-members alike. This has included equipment, staff, volunteers, facilities and financial resources. Without these inputs achievement of the College's objectives would have been futile.

The activities and processes underpinning the programmes delivered to the practitioners were designed to meet their needs and to potentially improve the public health. This has been attempted through teaching, distance learning, information exchange, seeking benefits for members such as indemnity insurance and also lobbying Government. COCA's educational and vocational outputs in Queensland can be measured by the number of seminars and conferences, the number of additional programs such as distance learning modules, the RACGP library access, the number of Journals and Newsletters supplied to members. This has been quite substantial for a relatively small organisation.

Outcomes can also be measured by the number of practitioners who have become members, the satisfaction ratings of attendees at seminars and conferences, the attraction of senior practitioners from within the State to become involved at organisational level.

Like all continuing education providers COCA aims (through its educational programs) to improve the participants knowledge and skills and although these are often considered to be rather short-term outcomes, they may also lead to positive changes in behaviours and then hopefully changes in values, conditions and improvements in the public health.

Further research should concentrate on the objective measurement of these outcomes and in particular patient health outcomes.

There have been limitations to COCA development in Queensland. Limited resources have prevented any attempt at objective measurement of practice outcomes from COCA's continuing education programs. COCA's first 6-years in Queensland have been more about development of and growing the organisation within the State. Nevertheless, the programs have been well received by surveyed participants and it should be noted that practical change by practitioners is unlikely to occur in an unhappy continuing education recipient.

Another major limitation has been the limited number of chiropractic or osteopathic academics on the ground in Queensland; it is postulated that this may be due in part to the lack of an under-graduate program in the State.

## Conclusion

The chiropractors and osteopaths in Queensland are now better serviced in the area of continuing education, vocational programs and professional assistance. This has been achieved by the development and expansion of the Chiropractic & Osteopathic College of Australasia within the State. It is hoped that COCA's continuing emphasis on information transaction skills, multi-disciplinary fora, science and ethics will have a positive impact on public health. To continue to be successful COCA must constantly review its modus operandi in the State of Queensland.
